# Infant feeding, growth monitoring and the double burden of malnutrition among children aged 6 months and their mothers in KwaZulu‐Natal, South Africa

**DOI:** 10.1111/mcn.13288

**Published:** 2021-11-29

**Authors:** Siri Kaldenbach, Ingunn M. S. Engebretsen, Lyn Haskins, Catherine Conolly, Christiane Horwood

**Affiliations:** ^1^ Department of Global Public Health and Primary Care, Centre for International Health University of Bergen Bergen Norway; ^2^ Department of Paediatric and Adolescent Medicine Innlandet Hospital Trust Lillehammer Norway; ^3^ School of Nursing and Public Health, Centre for Rural Health University of KwaZulu‐Natal Durban South Africa

**Keywords:** anthropometry, breastfeeding, complementary feeding, growth monitoring, infant and child nutrition, malnutrition, South Africa

## Abstract

South Africa has a documented high prevalence of stunting and increasing obesity in children as well as obesity in adults. The double burden of malnutrition, which can be on an individual‐, household‐ or population level, has implications for both health and the economic development of a community and country. This paper describes a large‐scale survey (*N* = 774) of infant feeding, growth monitoring and anthropometry among mother and child pairs aged 6 months of age in KwaZulu‐Natal (KZN), South Africa, conducted between January and August 2017. Among children, a large increase in the prevalence of stunting and obesity was seen between birth and 6 months of age increasing from 9.3% to 21.7% and 4.0% to 21.0%, respectively. 32.1% of the mothers were overweight [body mass index (BMI): 25.0–29.9] and 28.4% had obesity grade 1 (BMI: 30–<40). Although most mothers (93%; 563/605) initiated breastfeeding, the introduction of other foods started early with 17.6% (56/319) of the mothers having started giving other fluids or food to their child within the first month. At 6 months 70.6% (427/605) children were still breastfed and 23.5% were exclusively breastfed. In addition, we found that length measurements were done less frequently than weight measurements between birth and 6 months, on average 2.2 (SD: 1.3) versus 5.8 (SD: 1.5) times. Moreover, there is a need for improvement of health worker training and understanding regarding anthropometric measurements when assessing malnutrition in children in the clinics. Early detection and improved infant feeding practices are key in preventing both stunting and obesity in children.

## Key messages

- The double burden of malnutrition is severe in KwaZulu‐Natal with high rates of maternal obesity and increasing prevalence of stunting and obesity among infants in the first 6 months of life.
- Breastfeeding and complementary feeding practices are suboptimal and additional support for breastfeeding is required in the post‐natal period and in the workplace to address breastfeeding challenges.
- Growth monitoring is inadequate in health facilities leading to many lost opportunities for early identification of malnutrition. Hence, anthropometric practices and interpretation of findings need to be strengthened.

## Graphical abstract

The double burden of malnutrition is prevalent in South Africa with high rates of obesity among mothers, and increasing stunting and obesity among children during the first 6 months of life. This is potentially driven by poor breastfeeding and complementary feeding practices. As a result, improved training and facilitation for safer nutritional practices are warranted.

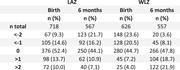

## 1 INTRODUCTION

Globally, there are 144 million children under the age of 5 years who are stunted and 47 million who are wasted, while 38.5 million are overweight or obese (World Health Organization, 2020). The term malnutrition encompasses undernutrition (wasting, stunting and underweight), inadequate vitamins or minerals, overweight and obesity according to the World Health Organization (WHO) (World Health Organization, 2020). When both over‐ and undernutrition exist at the same time within a country, community or individual, this is referred to as the double burden of malnutrition (DBM) (Popkin et al., 2020). Undernutrition in early life may lead to a higher risk of overweight later in life due to inappropriate or rapid weight gain when the nutritional conditions of the child improve (Martins et al., 2011). Moreover, the risk of DBM is increasing among people with low income in rural areas in low‐ and middle‐income countries (LMIC) due to a new nutrition reality with increasing consumption of ultra‐processed food leading to increasing prevalence of overweight and obesity in these communities (Wells et al., 2020).

DBM is a global concern. According to Popkin et al. (2020), this occurs when there is a rapid increase in the prevalence of overweight and obesity in LMIC, at the same time as these countries are experiencing a slower decline in the prevalence of undernutrition. Malnutrition in early life either undernutrition or, particularly obesity, can be the underlying cause of many non‐communicable diseases (NCDs) (Abarca‐Gómez et al., 2017). NCDs such as cardiovascular diseases, cancers, chronic respiratory disease, and diabetes, kill 41 million people each year, and there is a clear link between severe malnutrition in childhood and later development of cardiovascular disease, hypertension, and impaired glucose metabolism (Grey et al., 2021). In addition, rapid weight gain early in life has been shown to be a risk factor for later insulin resistance, obesity, and cardiovascular disease, particularly among formula‐fed infants (Singhal & Lucas, 2004). Therefore, it is important to reduce the risk factors associated with NCDs, which include unhealthy diets, malnutrition in early life and suboptimal breastfeeding practices.

Optimal breastfeeding is important for preventing malnutrition among infants and young children by providing the best source of energy and vitamins and by protecting children from childhood diseases that can adversely affect nutritional status. Infants who have been exclusively breastfed for 6 months have a decreased risk of diseases, such as gastrointestinal infections, pneumonia and hospitalisation compared with non‐exclusively breastfed or formula‐fed infants (Bahl et al., 2005; Horta et al., 2015; Ogbo et al., 2017). Sustained breastfeeding plays an important role at the time when complementary feeding is introduced, which frequently overlaps with linear growth faltering as children have to adapt to new tastes and textures (Wamani et al., 2006). Therefore, it is important to monitor growth, especially during the period when complementary foods are introduced.

The combination of women with overweight and children with stunting is the most common form of household‐level DBM (Popkin et al., 2020). Anthropometric measurements are used to categorise low birthweight, stunting (low height‐for‐age) or wasting (low weigh‐for‐height) during infancy or childhood to identify undernutrition (Wells et al., 2020). However, if lengths and weights are omitted or incorrectly measured or interpreted, malnutrition will be missed, and children will not receive the appropriate malnutrition support and management. To prevent long‐term morbidity, it is crucial that optimal feeding, particularly breastfeeding, is supported and that malnutrition, including obesity, is identified early through growth monitoring. In this paper, we describe a large‐scale survey of infant feeding, growth monitoring and DBM among mother/carer‐baby pairs at 6 months of age in KwaZulu‐Natal (KZN), South Africa.

## 2 METHODS

This study forms part of a larger study undertaken to estimate exclusive breastfeeding rates among 14 weeks old infants in KZN at two time points, known as KIBS1 (KwaZulu‐Natal Initiative for Breastfeeding support) and KIBS2 (Horwood et al., 2018, 2020). In this paper, we present the findings of a cross‐sectional survey conducted among mothers and caregivers of children aged 6 months (25‐31 weeks), which aimed to explore growth monitoring practices, anthropometry and feeding practices among 6‐month‐old children, and was conducted alongside the KIBS2 breastfeeding survey between January and August 2017.

### 2.1 Study setting

The study was undertaken in primary health care (PHC) clinics in KZN, one of the largest provinces in South Africa, with a population of over 11 million people (Stats SA, 2019). Free health care services are provided to mothers and children attending public health facilities in South Africa. PHC clinics provide the initial point of contact where maternal and child health services are provided, including antenatal, post‐natal and child health, nutrition, immunisation, and curative services.

A comprehensive schedule of immunisations is provided to all children in South Africa, including the first dose of the measles vaccine at 6 months. In addition, mothers are advised to bring their infants monthly for growth monitoring for the first 2 years of life (National Department of Health South Africa, 2019). In KZN over 80% of children are fully immunised at 1 year. However, severe malnutrition in children under 5 years of age remains high at 5.3/1000 children, and 29% of children are stunted. At the time of the study, infant mortality was estimated at 35 per 1000 live births in South Africa [National Department of Health (NDoH) et al., 2019].

### 2.2 Sampling

The sample size was calculated based on obtaining valid estimates for breastfeeding rates among children at 14 weeks for the KIBS2 study. Thirty clinics were randomly sampled, and the sample included clinics in all districts of the province. This survey was conducted alongside KIBS2 and caregivers attending with 6‐month‐old children were recruited for the duration of the KIBS2 study period but were not part of the KIBS2 study.

All mothers or caregivers aged 15 years or above who attended the participating clinics with a child aged 6 months (25–31 weeks) were eligible to participate in the study. The 6 months age was chosen to coincide with the time when children attend for measles immunisation, which presented an opportunity to reach children in a narrow age band. Non‐maternal caregivers answered a subset of relevant questions.

### 2.3 Data collection

Exit interviews were conducted after completion of the clinic visit by trained fieldworkers in the local language (English or isiZulu) using structured questionnaires (Supporting Information File 1). Background data, such as age, education level and household setting, including access to water and electricity were asked.

Mothers and non‐maternal caregivers were asked questions about feeding practices since birth and other feeding practices such as whether any other food or fluids were given to the child together with, or instead of breastmilk. Current feeding practices were assessed using a 24‐h food and fluids recall. Moreover, mothers and non‐maternal caregivers were asked about their knowledge and attitudes towards breastfeeding with statements and questions. The questions were a series of true/false (T/F) questions constructed in collaboration with the Nutrition Directorate, Department of Health in KZN. These included the following statements: breastfed babies have less diarrhoea (T); a mother who feels the baby is not getting enough breastmilk should top up with formula milk (F); infant formula contains all the ingredients found in breastmilk (F).

Patient‐held records for the children [Road to Health Card (RTHC)] were reviewed by fieldworkers and all anthropometric data (length and weight measurements) recorded on the RTHC since birth until the day of data collection were captured, together with the date of recording. The mother's current height and weight were measured and recorded at the site.

### 2.4 Ethical considerations

Ethical approval was obtained from the Biomedical Research Ethics Committee at the University of KwaZulu‐Natal (BE064/14) and from the KZN Department of Health. All participants provided written informed consent. Confidentiality and anonymity were assured through the allocation of study numbers. To ensure all mothers of young children were able to participate, ethical approval explicitly allowed the inclusion of younger mothers aged 15–17 years. Permission to undertake the study was obtained from the KZN Department of Health, district managers in all districts, and facility managers in participating clinics.

### 2.5 Data management and analysis

Data was captured on handheld android tablets and uploaded to a central server in real time. Extensive quality control checks were carried out by trained study staff.

Data were cleaned and analysed in Stata 16.0 (StataCorp, 2019). Anthropometric data was cleaned in two stages. First, as anthropometric data was captured from the RTHC, inter‐ and intra‐rater reliability could not be assessed. Therefore, if errors in the recording of the data of children were identified this data was removed from the data set. Errors of recording occurred when the value recorded was incompatible with a child's length or weight. The following numbers of children were removed: seven for birthweight, two for birth length, 14 for weight at 6 months and 45 for lengths at 6 months.

Second, the anthropometric data were cleaned based on attained *z*‐scores from the WHO Child Growth Standards. Statistical analysis was undertaken using the Stata command ‘zscore06’ to calculate the different *z*‐scores; Length‐for‐age z‐score (LAZ) and weight‐for‐length z‐score (WLZ). Measurements were flagged at the following criteria

1. LAZ < −6 or >6;
2. WLZ < −5 or >5 or
3. WLZ > WLZ > 3 and LAZ < −3.

Measurements were set to missing if one or more of these extreme values existed after individually assessing them. The following numbers of children with extreme values were excluded: two for LAZ at birth; seven for LAZ at 6 months; 22 for WLZ at birth; 17 for WLZ at 6 months.

There was a wide variation in the quality and number of measurements across clinic visits from birth to 6 months. The study team's presence at the site is a likely reason for an increased number of weight and length measurements performed at the time of the interview. However, to display the difference in weight and length outcomes, all recorded measurements were included. Therefore, this resulted in different denominators for calculations regarding length and weight, such as LAZ and WLZ.

Descriptive statistical analyses were undertaken to describe the characteristics and distribution of the population. Categorical data are presented as percentages while continuous data are presented as means with standard deviations and confidence intervals.

Multi‐variable analysis was used to investigate potential risk factors with the dependent variables LAZ and WLZ with cut‐offs at <−2 and >2 *z*‐scores, respectively. LAZ < −2 indicates stunting and WLZ > 2 indicates overweight. Selected variables were based on the UNICEF Conceptual framework on young child malnutrition from 1991 (United Nations Children's Fund, 1991). The selected variables were gender, birthweight, household information, reported breastfeeding practices for the first 6 months and current breastfeeding practices (assessed through 24 h recall), mother's age, mother returning to school, mother's height and HIV status. These were all included in the final model because of potential confounding factors. Only the adjusted OR with 95% CI analysis is presented in the results.

## 3 RESULTS

Seven hundred and seventy‐four interviews were conducted with caregivers of children aged 6 months attending PHC clinics between January and August 2017. Most children attended the clinic with their mother (605/774; 78.2%) but some children attended with other caregivers, referred to as non‐maternal caregivers (169/774; 21.8%). Among the non‐maternal caregivers, most were relatives of the child. Non‐maternal caregivers reported that the reason for the mother's absence was either because: the mother was at work (102/169; 60.3%), the mother was at school (53/169; 31.4%), the mother was unwell (2/169; 1.1%) or other reasons (not specified) (9/169; 5.3%). Demographic information about all caregivers is shown in Table 1; however, some questions were not asked to non‐maternal caregivers.

**Table 1:** Participants' demographic information

	Mothers (*n* = 605)		Non‐maternal caregivers (*n* = 169)		Total (*n* = 774)	
Categorical data	*n*	%	*n*	%	*n*	%
All caregivers						
Female child	301	49.8	85	50.3	386	49.9
Living in a rural or urban area						
Rural	443	73.2	131	77.5	574	74.2
Urban	162	26.8	38	22.5	200	25.8
Mother lives with the baby	588	97.2	107	63.3	695	89.8
Mothers only						
Maternal age						
15–17	20	3.3				
≥18	584	96.5				
Missing age	1	0.2				
Child has siblings	370	61.2				
Mother in relationship with father	538	88.9				
Mother HIV‐positive	238	39.3				
Mother's highest school grade						
0–7	42	6.9				
8–12	563	93.1				
Mother returned to work or school	125	20.7				
Household information						
Private water access^a^	337	55.7	98	58.0^b^	435	56.2
Electricity in house	513	84.8	138	81.7	651	84.1
Uses electricity for cooking	430	71.1	123	72.8	553	71.5

^a^ Private water access refers to either piped water in the yard or piped inside.

^b^ 7.1% had missing information about water access.

### 3.1 Infant feeding practices at 6 months among children brought by the mother

Mothers (*N* = 605) were asked about breastfeeding practices over the first 6 months of the child's life, including how long they breastfed and when they first gave the child other food or fluids. Most of the mothers (563/605; 93.1%) had initiated breastfeeding, with 6.9% (42/605) of the mothers reporting that they had never breastfed. Among the 563 mothers who reported initiating breastfeeding, most (319/563; 56.7%) reported that they had started giving other fluids or food to their child in addition to breastmilk. This includes 17.6% (56/319) who reported starting other food or fluids in the first month of the child's life. Among all children attending with their mother, 70.6% (427/605) were still being breastfed at 6 months, with 23.5% (142/605) of the children still being exclusively breastfed.

At the time of the interview, 22.5% (136/605) of mothers reported they had stopped breastfeeding. Reasons for stopping breastfeeding included: returning to work (45/136; 33.1%); breastfeeding challenges (33/136; 24.3%); mother's health (including HIV) (20/136; 14.7%); health worker advised not to breastfeed (8/136; 5.9%); family advised not to breastfeed (3/136; 2.2%) or other reasons (27/136; 19.8%).

Among 319 mothers who had given other fluids or foods to their children while continuing to breastfeed, 314 (314/319; 98.4%) reported the reason for this. Reasons given included mainly the mothers' experiences with or perceptions about breastfeeding (including not enough milk, baby crying or previous bad experience) (108/319; 33.9%). Other reasons given were returning to work or school (82/314; 26.1%), or health workers advising to add other foods (44/314; 14.0%). Other foods or fluids given included: water; infant formula; condensed or evaporated milk, powdered or fresh animal milk; yoghurt, amaasi (fermented milk) or thin porridge; traditional medicine.

Mothers displayed a positive attitude and good knowledge of breastfeeding with over 75% of the mothers having 9 or more correct answers out of a total of 12 questions/claims about breastfeeding, with 23.1% having 11 or more correct answers.

### 3.2 Complementary feeding

All caregivers (*n* = 774) were asked if they had been with the baby over the past 24 h and could provide information about all the food and fluid the child had consumed in the previous 24 h, and 662 were able to report on this. Among these 422/662 (63.7%) of the children were still breastfed (mixed and exclusive) at the time of the interview.

Of all children, 48.3% (320/662) had received yoghurt or amaasi (fermented milk) and 43.6% (289/662) had received infant formula in the past 24 h. Among those children no longer being breastfed 98.3% (236/240) received infant formula. Other foods typically given included commercial baby cereal, bread, rice, porridge, or other grains. Among children who were still breastfed, 45.8% (193/422) received yoghurt or amaasi, 32.9% (139/422) received commercial baby cereal and 25.8% (109/422) received bread, rice, porridge or other grains. None of the caregivers reported that the child had received other recommended complementary feeds like egg, mashed vegetables or fruit.

### 3.3 Growth monitoring

**Figure 1:**
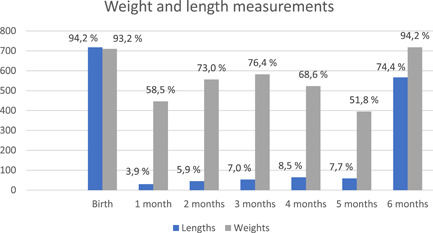
Weight and length measurements according to age (*N* = 772)

Among all participants, 772/774 brought the child's RTHC with them on the day of the visit. To assess the coverage of growth monitoring among participants, we determined the proportion of children who had their weight and length measured at each month of age. This was based on recordings of length and weight on the RTHC. The proportion of children who had recorded weight and length measurements at each month is presented in Figure 1 (*n* = 772).

The average number of weight measurements recorded on the RTHC for each child between birth and 6 months was 5.8 (SD: 1.5), and the average number of length measurements was 2.2 (SD: 1.3). Most children had their length and weight recorded at birth and at 6 months. The number of length measurements recorded between birth and 6 months was less than the number of weight measurements recorded. In this time period, 70.2% of the children had the recommended 6 weights recorded (i.e. at birth and monthly thereafter) while only 3.8% had more than 6 or more lengths recorded (Table 2).

**Table 2:** Frequency of growth monitoring among participating children as recorded on the RTHC

	Number of children (*n* = 774), *N* (%)
Number of times the weight was recorded on the RTHC between birth and 6 months	
0–1	22 (2.8)
2–3	32 (4.1)
4–5	177 (22.9)
>6	543 (70.2)
Number of times the lengths were recorded on the RTHC between birth and 6 months	
0–1	176 (22.7)
2–3	494 (63.8)
4–5	75 (9.7)
>6	29 (3.8)

Abbreviation: RTHC, Road to Health Card.

### 3.4 Mother's anthropometry

Of the mothers who attended the clinic (*n* = 605), 545 mothers had their weight recorded and 529 had their height recorded. Body mass index (BMI) was calculated for 507 mothers where both weight and height were recorded on the day of the visit, excluding extreme values and outliers. Among these mothers 3.7% (19/507) were underweight (BMI: <18.5), 35.7% (181/507) were normal weight (BMI: 18.5–24.9), 32.1% (163/507) were overweight (BMI: 25.0–29.9) and 28.4% (144/507) of the mothers had obesity grade I (BMI: 30–<40). The average BMI of the mothers was 27.3 (SD: 6.2).

### 3.5 Infants' anthropometry

Table 3 provides information on LAZ and WLZ at birth and 6 months for the various cut‐off points from <−2 to >2. The total numbers differ by column due to inconsistent measurements (children having either no length or no weight at the measurement time point) but also due to the WHO cut‐offs explained in the methods. Thus, the denominator changes according to the data available. The reason for keeping the different denominators was to illustrate the difference in measures (lengths and weights) taken at the clinics as shown in Figure 1. Nevertheless, the tables show strikingly large percentages both at birth and at 6 months for the cut‐off below −2 and above 2. Moreover, 53/555 (9.5%) of the children were both stunted and overweight in the sample at 6 months. There was one child who was both stunted and wasted at 6 months.

**Table 3:** Prevalence of stunting, wasting and excess weight at birth and 6 months

	LAZ		WLZ	
	Birth	6 months	Birth	6 months
	*n* (%)	*n* (%)	*n* (%)	*n* (%)
*n* total	718	567	626	557
<−2	67 (9.3)	123 (21.7)	148 (23.6)	20 (3.6)
<−1	105 (14.6)	92 (16.2)	128 (20.5)	45 (8.1)
0	376 (52.4)	250 (44.1)	280 (44.7)	266 (47.8)
>1	98 (13.7)	62 (10.9)	45 (7.2)	104 (18.7)
>2	72 (10.0)	40 (7.1)	25 (4.0)	122 (21.9)

Table 4 displays the categories of LAZ (stunting and normal) and WLZ (normal and overweight) at 6 months by birthweight categories. There were 482 children who had all the information available; birthweight, weight and length at 6 months.

**Table 4:** Prevalence of obesity and stunting among children at 6 months according to birthweight (BW)

	LAZ at 6 months					WLZ at 6 months				
	Stunting at 6 months		Normal		Total	Normal at 6 months		Excess at 6 months		
	*n*	%	*n*	%	*n*	*n*	%	*n*	%	Total
BW < 2.5 kg	14	33.3	28	66.7	42	35	83.3	7	16.7	42
Normal BW 2.5–4 kg	75	17.4	356	82.6	431	332	77.0	99	23.0	431
High BW > 4 kg	0	0.0	9	100.0	9	4	44.4	5	55.6	9
Total	89	18.5	393	81.5	482	371	77.0	111	23.0	482

Abbreviations: LAZ, length‐for‐age *z*‐score; WLZ, weight‐for‐length *z*‐score.

According to the adjusted logistic regression (Table 5), we found that the odds ratio (OR, 95% confidence interval) for low birthweight (below 2500 g) was a risk factor for stunted growth at 6 months (OR: 3.64, 1.92; 6.88). Female sex seems protective with respect to stunting at 6 months (OR: 0.64, 0.38; 1.09), but this value was not statistically significant. There were no other associations found with stunting at 6 months. Moreover, no clear factors were associated with obesity at 6 months.

**Table 5:** Adjusted association between stunting and selected risk factors, odds ratio [OR and 95% confidence interval (CI) reported]

	OR	95% CI
Female sex	0.64	0.38, 1.09
Low birthweight (<2500 g)	3.64	1.92, 6.88
Still breastfeeding	1.21	0.63, 2.32
HIV‐positive	1.16	0.66, 2.03
Having siblings	0.81	0.46, 1.42
Mother's age <18 years	0.48	0.06, 4.20
Mother's length	0.06	0.00, 1.34
Mother returned to work/school	0.91	0.43, 1.91
Access to private water	1.14	0.67, 1.92

Abbreviations: DBM, double burden of malnutrition; KIBS, KwaZulu‐Natal Initiative for Breastfeeding Support; LAZ, length‐for‐age *z*‐score; LMIC, low‐and middle‐income country; NCD, non‐communicable disease; PHC, primary health care; RTHC, Road to Health Card; SA, South Africa; SD, standard deviation; WAZ, weight‐for‐age *z*‐score; WHO, World Health Organization; WLZ, weight‐for‐length *z*‐score.

## 4 DISCUSSION

We found that DBM was prevalent in KZN, with the majority of mothers being overweight or obese, and many children developing stunting in the early months of life. Breastfeeding practices were suboptimal and exclusive breastfeeding was uncommon with many mothers adding other food and fluids to the infant's diet even in the first month of life. Although in our study we did not find a significant association between breastfeeding and stunting, other studies support this association and it is likely that inadequate breastfeeding contributed to the high rates of stunting (Black et al., 2013; Campos et al., 2020). Further, our findings suggest that complementary feeding is inadequate at the time of most vulnerability for the development of malnutrition, and children did not get the recommended variety of nutritious complementary food. This occurs against a background of inadequate growth monitoring in clinics, which hinders the early identification of malnutrition so that preventive interventions cannot be implemented. Our findings suggest that there are multiple contributing factors to the high rates of chronic malnutrition and the DBM in SA.

Although most mothers initiated breastfeeding and many were still breastfeeding their children at 6 months, poor infant feeding practices were observed. First, there was a tendency to add other foods or fluids very early. Early introduction of complementary feeds has shown to be a risk factor for faltering growth (Martins et al., 2011), which might lead to overweight and obesity later in life (Ramokolo et al., 2015). Moreover, children who are stunted, wasted or underweight have an increased risk of death from diarrhoea, pneumonia and other infectious diseases (Black et al., 2013). Identification of behavioural and structural barriers to delaying the introduction of other foods is needed to improve exclusive breastfeeding practices in this area. In particular, the common reasons for adding other foods included mothers' experiences with or perceptions about breastfeeding and returning to work or school. Therefore, stronger support is needed for breastfeeding not only at the time of delivery but also for mothers experiencing breastfeeding challenges after returning home or when returning to work or school. Community health workers are deployed in communities in South Africa and are ideally placed to support breastfeeding at the household level. Similar reasons and findings have also been detected in other sub‐Saharan countries (Maonga et al., 2016; Masuke et al., 2021). Although there is strong support for breastfeeding provided during antenatal care and most mothers intended to breastfeed, most women fail to breastfeed exclusively or sustain breastfeeding (Horwood et al., 2020; Richard et al., 2021). There is a need to develop and evaluate strategies for sustaining breastfeeding in our setting.

According to the SA child health guidelines (National Department of Health South Africa, 2019), recommended complementary feeds are soft/mashed foods, including cereals, fruit and vegetables and family foods. The key message is that complementary foods should be energy‐rich and micronutrient‐sufficient to prevent faltering growth when shifting from exclusive breastfeeding to breastfeeding and complementary foods. This study found that none of the caregivers provided mashed vegetables and fruits. There may be a need to do further nutritional research on complementary diets using locally available foods and strategies put in place to improve current practices. Mothers need practical guidance on how to feed their children, both the timing, what food to give, and how much, particularly if they are no longer breastfeeding. A controversial step could involve investigating alternatively affordable, acceptable and homemade diets given to children from earlier ages than 6 months. Rather than the single message focusing on exclusive breastfeeding to 6 months, which may exclude the high proportion of mothers unable to achieve exclusive breastfeeding for the recommended period. Exclusive breastfeeding rates have improved in SA (Horwood et al., 2020; Jackson et al., 2019; NDoH et al., 2019) but the transition from breastfeeding to complementary feeding has not received sufficient attention. A multi‐sectoral analysis taking the knowledge of parents and health workers into account while considering whether what is recommended can be acceptable and available in all its dimensions (access, cost and distribution) is needed.

Weight measurements were performed more often than length measurements even among children whose weight was recorded at the clinic visit. To monitor growth effectively and to correctly identify malnutrition, both measurements are important as weight might fluctuate more than lengths. Given that 21.7% of the children were stunted at 6 months, it is likely that better growth monitoring could have detected growth faltering earlier, particularly in infants with low birthweight who are particularly at risk. Length measurements can be challenging to perform in small children who are not able to stretch completely and health workers may depend on the mother or another person in the room to assist, and a length board should be used (World Health Organization, 2008). Measuring length can be demanding, inconvenient and time‐consuming during busy clinic days. It is therefore important to communicate the importance of LAZ and WLZ and to train health workers in how to perform them.

Another possible reason for failure to measure length could be that the health workers were not adequately competent to calculate LAZ or WLZ and to interpret the results. Weight‐for‐age z‐score does not discriminate between ponderal growth (as seen with WLZ) and growth performance (including growth faltering as seen with LAZ). There are numerous digital tools available to overcome this hurdle, which are currently unavailable but could be implemented in clinics with training. For instance, the Emergency Nutrition Assessment (ENA) is an analytical programme aimed to help health workers to do anthropometric assessments (SMART METHODOLOGY, 2020). Even though ENA is not dependent on WI‐FI to calculate these measures, increasing Internet coverage gives rise to other possible options in terms of technology. Irrespective of advanced technologies, there is a need to improve length measurements timeliness and corresponding responses. This is particularly important for children with a low birthweight, who should be identified as at risk. CHWs have a role to play in supporting growth monitoring in the community, particularly for at‐risk children. Nevertheless, training of staff and teaching of the importance of length in assessing malnutrition needs to be improved overall.

Household‐level DBM is clearly present with high levels of overweight and obesity among mothers, where at the same time high levels of stunted and overweight children were observed. Improved management of moderate malnutrition and identification of malnutrition at the household levels for both mother and child is therefore important, and nutrition advice may need to focus more on the mother‐infant dyad rather than a more traditional focus on infants. How mothers may benefit from breastfeeding is often under communicated.

### 4.1 Strengths and limitations

The major strength of this paper is the large representative sample from KwaZulu‐Natal, which is a large province in South Africa. Moreover, according to the SANHANES (South Africa National Health and Nutrition Examination Survey—1), stunting is similar in all provinces and it is therefore likely that these findings are similar across South Africa (Shisana et al., 2013). This cross‐sectional study also took advantage of the available operational data to get an overview of monitoring practices. Of course, it is a weakness in terms of reporting growth that the growth data was not complete and that multiple measurements had to be excluded because the quality was so poor, which suggests that measurements were inaccurate. Given that most participants were from rural areas, it is possible that urban clinics would have performed better. On the other hand, the operational data illudes to a naturalist description of monitoring, which should stimulate further discussion, updated guidelines, and hopefully improved practices.

A limitation of the study was having a long recall period of up to 6 months, which can provide recall bias as mothers and non‐maternal caregivers might have difficulty recalling exactly what the infant was fed. There might also possibly have been social desirability bias on how the mothers and non‐maternal caregivers answered the questions.

## 5 CONCLUSION

This study highlights that length and weight measurements taken during the first 6 months of a child's life are inadequate in both quantity and quality, based on the number of outlying values. This limits the effectiveness of growth monitoring and assessment of nutritional status. In addition, high rates of both stunting and overweight among children and overweight and obesity in mothers warrants better growth monitoring and detection of malnutrition. Moreover, infant feeding practices, including breastfeeding practices and complementary feeding may need far deeper understanding for improving both guidelines and health services.

## CONFLICT OF INTERESTS

The authors declare that there are no conflict of interests.

## AUTHOR CONTRIBUTIONS

Christiane Horwood and Lyn Haskins performed the research and designed the research study. Siri Kaldenbach and Catherine Conolly analysed the data. All authors (Siri Kaldenbach, Ingunn M. S. Engebretsen, Lyn Haskins, Catherine Conolly and Christiane Horwood) wrote the paper. All authors approved the final version of the manuscript.

## Supporting information

Supporting information.

## Data Availability

The data and study tools that support the findings of this study are available from the Centre for Rural Health and will be made available on reasonable request.
