# Supplementary material for: Infant feeding, growth monitoring and the double burden of malnutrition among children aged 6 months and their mothers in KwaZulu‐Natal, South Africa
Source: Matern Child Nutr. 2021 Nov 29;18(1):e13288. doi: 10.1111/mcn.13288 (PMC8710097; doi:10.1111/mcn.13288)
Supplement: Supplementary file 1 — Supporting information. [file MCN-18-e13288-s001.pdf]

KWAZULU-NATAL INITIATIVE FOR BREASTFEEDING SUPPORT

SECTION 1: ADMINISTRATION (A)												
**A1** 1.1	District name											
**A3** 1.2	Clinic name											
**A5** 1.3	Date of interview	D	D	M	M	Y	Y	Y	Y	**A6** 1.4	Interviewer:	

SECTION 2: SCREENING QUESTIONS (S)			
**S1** 2.1	Have you ever taken part in the KIBS breastfeeding study before? **Uke waba ingxenye yocwaningo lokuncelisa (i-KIBS)?**	1 = Yes ➔ ***End interview***	0 = No
**S2** 2.2	What is the baby's date of birth? **Luthuni usuku lokuzalwa lomntwana?**	D D M M Y Y Y Y	

14week survey includes infants aged 91-111days:
Is baby below 13 weeks old ➔ End interview
Is baby between 13 weeks (91 days) and 15 weeks (111 days) old ➔ continue with interview
Is baby older than 15 weeks (111 days) old ➔ check next age category
6months survey includes infants aged 6 completed months up to 7 months:
Is baby between 15 weeks (111 days) and less than 6 months old ➔ End interview
Is baby 6 months old (25weeks) but less than 7 months (31weeks) old ➔ continue with interview
Is baby older than 7 months old ➔ End interview

**S3** 2.3	Is the baby in the 13-15week age group or in the 6-7month age group? **Ingabe umntwana unamaviki awu-13 kuya ku-15 noma unezinyanga ezingu-6 kuya ku-7 ubudala?**	1= 13-15 weeks	2 = 6-7 months
**S6** 2.4	What is your date of birth? **Luthini usuku lakho lokuzalwa?**	D D M M Y Y Y Y	
**S7** 2.5	How old are you? **Uneminyaka emingaki?**	……………………….. years	

IF AGED 15 YEARS OR ABOVE DO INFORMED CONSENT
IF AGED BELOW 15YEARS➔ END INTERVIEW

S8 2.6	Was consent obtained? **Itholiwe imvumo?**	1 = Yes
		0 = No, participant refused to participate ➔ ***End Interview***
		2= Participant ineligible ➔ ***End Interview***

Fill in the tracking form now

S9 2.7	**INTERVIEW NUMBER**	**C**						
**S10** 2.8	What is the sex of the baby? **Buthini ubulili bomntwana?**	1 = Male		0 = Female				
**S11** 2.9	OBSERVE: Population group of the respondent (mother or caregiver)	1 = African	2 = coloured	3 = Indian	4 = White	5= Other		

**S13**<br>2.10	Does the mother live in the same household as the child?<br>**Ingabe umama womntwana uhlala endlini eyodwa nomntwana?**<br>(sleeps in the same household as baby at least four nights a week)	1 = Yes ➔ ***Skip to S15***	0 = No
**S14**<br>2.11	In whose household does the baby live?<br>**Uhlala emzini kabani umntwana?**	1= with mothers family<br>2= with fathers family<br>3= with non-relative caregiver	
**S15**<br>2.12	What is the reason for you attending the clinic today?<br>**Isiphi isizathu sakho esenza ukuba uze emtholampilo namuhla?**<br>**More than one response allowed: mark any options that applies**	1= immunisation/weighing/well child ***Uzogoma/uzokala/umntwana uphilile***<br>2= child is sick ***Umntwana uyagula***<br>3= services for the mother ***umama oze emtholampilo***<br>4= other ***okunye***	
**S16**<br>2.13	I would like to ask you some questions about this baby. What is the name of the baby? I only need this information so that I can call this baby by name.<br>**Ngicela ukubuza imibuzo ngalomntwana. Ubani igama lomntwana? Ngidinga ukwazi igama lomntwana ukuze ngimubize ngalo kulengxoxo yethu.**	………………………………	
**S17**<br>2.14	Are you the mother of the baby?<br>**Ingabe uwena umama walomntwana?**	1= Yes<br>➔ ***Skip to M0***	0 = No

**SECTION 3: NON MATERNAL CAREGIVERS (NM)**			
**NM1**<br>3.1	What is your relationship to (name)?<br>**Buthini ubudlelwano bakho nomntwana?**	1 = Grandmother ***ugogo***<br>2 = Other relative ***Ezinye izihlobo zomndeni***<br>3 = Father ***ubaba womntwana ➔ Skip to NM4***<br>4 = Non relative care taker ***umuntu ogada umntwana ongasisona isihlobo somndeni***	
**NM3**<br>3.2	Are you receiving any payment for looking after (name)?<br>**Ingabe uyaholelwa ukuthi ugade u (gama)?**	1 = Yes	0 = No
**NM4**<br>3.3	Do you live in the same household as the baby?<br>**Ingabe uhlala endlini eyodwa nomntwana?**	1 = Yes	0 = No
NM2<br>3.4	Is the mother of (name) still alive?<br>**Ingabe usaphila umama womntwana?**	1 = Yes	0 = No ➔ ***Skip to NM7***

**NM5**<br>3.5	Where is the mother today?<br>**Ukuphi umama namhlanje?**	1= working/**uyasebenza**<br>2= at school/**usesikoleni**<br>3= unwell/**akaphilile**<br>4= not present for another reason/**akekhona ngenxa yesinye isizathu**		
**NM6**<br>3.6	Is (name) currently being breast fed?<br>**Njengamanje uyalinceliswa ibele u (gama)?**	1 = Yes<br>→***Skip to NM8***	0 = No	-1 = Do not know
**NM7**<br>3.7	Has (name) ever been breastfed?<br>**Ingabe u(gama) wake wanceliswa ibele?**	1 = Yes	0 = No	-1 = Do not know
**NM8**<br>3.8	What food or fluid are you giving (name) today while you are at the clinic?<br>**Ikuphi ukudla noma okusaketshezi okupha u (gama) njengoba nise mtholampilo namhlanje?**	1= Breastmilk<br>***ubisi lwebele***	2= Other<br>***okunye***<br>→***Skip to NM10***	
**NM9**<br>3.9	Has (name) ever been given any other foods or fluids other than breast milk?<br>**Kungabe u (gama) wake wanikezwa ukudla noma okusaketshezi ngaphandle kobisi lwebele?**	1 = Yes	0 = No	-1 = Do not know
**NM10**<br>3.10	Are you able to report about the household where (name) is living?<br>**Ungakwazi ukungitshela mayelana nekhaya lapho u (gama) ahlala khona?**	1= yes<br>→***Skip to H5***	0= no<br>→***Skip to IF11***	

**SECTION 4: MOTHERS INFORMATION (M)**			
Now I would like to ask some questions about you.<br>**Ngicela ukukubuza imibuzo ngawe.**			
**M0**<br>4.1	What is your (most recent) relationship status?<br>**Isiphi isimo sobudlelwane okuso njengamanje? (most recent)**<br><br>Select most appropriate response	1= Married<br>**ushadile**<br>2=separated/divorced/widowed (not in a relationship currently)<br>**Nihlukene/unesihlukaniso/umfelokazi (awunabo ubudlelwano njengamanje)**<br>3= In a relationship, living with partner **Nginabo ubudlelwano futhi ngihlala naye umuntu engizwana naye**<br>4= In a relationship, not living with partner<br>**Nginabo ubudlelwano kodwa angihlali naye umuntu engizwana naye**<br>5 = Single<br>**anginabo ubudlelwano**	
**M1**<br>4.2	Do you have any other biological children who are older than (name)?<br>**Unabo abanye abantwana abazalwa nguwena ngaphandle kuka (gama)?**<br>*Do not include a twin of the index child*	1 = Yes	0 = No → ***Skip to M4***
**M2**<br>4.3	How many biological children do you have?<br>**Bangaki abantwana abazalwa uwena?**<br>*Include all liveborn children*	……………… children	

<table>
<tr><td rowspan="3">M3
4.4</td><td rowspan="3">How did you feed your last child for the first three months?
Wayedlani umntwana wakho olanywa umagcino ezinyangeni ezintathu ezelwe?</td><td colspan="3">1= Breastmilk only (ubisi lwebele kuphela)</td></tr>
<tr><td colspan="3">2=Breastmilk and other food or fluids (ubisi lwebele kanye nokunye ukudla noma okusaketshezi)</td></tr>
<tr><td colspan="3">3=No breastmilk, only other foods or fluids (akancelanga ubisi lwebele, wadla okunye ukudla noma okusaketshezi)</td></tr>
<tr><td>M4
4.5</td><td>Is the father of (name) still alive?
Usaphila ubaba womntwana?</td><td>1 = Yes</td><td>0 = No
➔ Skip to M7</td><td>-1 = Do not know
➔ Skip to M7</td></tr>
<tr><td>M5
4.6</td><td colspan="2">Are you still in a relationship with the father of (name)?
Ngabe usenabo ubudlelwano nobaba womntwana?</td><td>1 = Yes</td><td>0 = No
➔ Skip to M6_1</td></tr>
<tr><td>M6
4.7</td><td colspan="2">Are you staying in the same house as the father of (name) most nights?
Ngabe uhlala naye ubaba womntwana?

4 nights or more a week</td><td>1 = Yes</td><td>0 = No</td></tr>
<tr><td>M6_1
4.8</td><td colspan="2">Has the father provided any money or bought things to care for the baby in the past one month?
Ingabe ubaba womntwana ukewakusiza (ngemali noma athenge izinto ezinjenge zimpahla zokugqoka, amanabukeni noma ubisi lwethini) ngokunakekela u (igama) kulenyanga edlule?</td><td>1 = Yes</td><td>0= No</td></tr>
<tr><td rowspan="3">M6_2
4.9</td><td rowspan="3">How often does the father contribute money or other items to care for (name)?
Ingabe ubaba wengane ukunika kangaki imali noma ezinye izinto ukuze unakekele u (igama)?</td><td colspan="3">1= never/
akaze</td></tr>
<tr><td colspan="3">2= sometimes/
kuqabukela</td></tr>
<tr><td colspan="3">3= regularly (every week or every month)/
ujwayele (njalo ngamasonto noma njalo ngenyanga)</td></tr>
<tr><td rowspan="6">M7
4.10</td><td rowspan="6">Who else is staying in the house with you?
Ubani omunye ohlala nawe?

More than one option allowed</td><td colspan="3">1 = Grandmother of the child
ugogo womntwana</td></tr>
<tr><td colspan="3">2 = Your siblings
abafowenu nodadewenu</td></tr>
<tr><td colspan="3">3 = Other family members
amanye amalungu omndeni</td></tr>
<tr><td colspan="3">4 = Your other biological children abanye abantwana abazalwa yimi</td></tr>
<tr><td colspan="3">5 = Non family members
amanye amalungu ekungasiwona omndeni</td></tr>
<tr><td colspan="3">6= stays alone (with or without the baby)</td></tr>
<tr><td>M8
4.11</td><td>What was the highest grade you passed in school?
Iliphi ibanga oliphumelele esikoleni?</td><td>0 = None/ never attended school</td><td>1 = Grade 1- 11

Write no of highest grade completed/passed</td><td>2= Grade 12

➔ skip to M17</td></tr>
</table>

**M9**<br>4.12	Are you attending school currently or planning to go back to school in the future?<br>**Ingabe usesikoleni njengamanje noma uhlele ukubuyela esikoleni esikhathini esizayo?**		1 = Yes	0 = No
**M10**<br>4.13	Have you been back to school since the baby was born?<br>**Ingabe wabuyela yini esikoleni emuva kokuba u (gama) ezelwe?**		1 = Yes	0 = No<br>**➔SKIP to M17**
**M11**<br>4.14	What food is given to the baby while you are at school?<br>**Udlani umntwana wakho uma usesikoleni?**<br><br>***More than one option allowed***<br>***Impendulo edlule kweyodwa ivumelekile***	1= breastfeed (you feed the baby from the breast)<br>**uncela ubisi lwebele (uncelisa umntwana ebeleni)**		
		2= expressed breastmilk (another person feeds the baby with expressed breastmilk)<br>**Ubisi lwebele olukhanyiwe (omunye umuntu oncelisa umntwana ubisi lwebele olukhanyiwe)**		
		3= Formula milk/ other food or fluids **ubisi lwebhodlela/ okunye ukudla noma okusaketshezi**		
**M17**<br>4.15	Have you done any paid work since the baby was born?<br>**Kukhona la oke wasebenza khona wathola imali emva kokuthola umntwana?**		1 = Yes	0 = No ➔ ***skip to M30***
**M18**<br>4.16	How are you feeding your child while you are at work?<br>**Udlani umntwana ngesikhathi usemsebenzini?**<br><br>***More than one option allowed***<br>***Impendulo edlule kweyodwa ivumelekile***	1= breastfeed (you feed the baby from the breast)<br>**uncela ubisi lwebele (uncelisa umntwana ebeleni)**		
		2= expressed breastmilk (another person feeds the baby with breastmilk)<br>**Ubisi lwebele olusengiwe (omunye umuntu oncelisa umntwana ubisi lwebele olukhanyiwe)**		
		3= Formula milk/ other food or fluids<br>**ubisi lwebhodlela/ okunye ukudla noma okusaketshezi**		
**M29**<br>4.17	Who **usually** looks after your baby when you are at school or work?<br>**Ubani ovamise ukugada umntwana uma useskoleni noma emsebenzini noma ukude naye?**<br><br>***ONLY ONE RESPONSE ALLOWED***<br>***KUVUNYELWE IMPENDULO EYODWA KUPHELA***	1= take baby to work/school<br>**ngihamba naye umntwana mangiya emsebenzini noma esikoleni**		
		2= relative or unpaid carer<br>**uhlala nezihlobo noma kukhona omgadayo ongakhokhelwa**		
		3= crèche or paid carer **uya enkulisa noma khona omgadayo okhokhelwayo**		
		4= never been away from the baby<br>**angikaze ngibe kude nomntwana**		
		5= other<br>**okunye**		
M30<br>4.18	Have you ever given (baby name) expressed breastmilk while you are away from him/her?<br>**Useke waphiwa umntwana ubisi lwebele olukhanyiwe ngesikhathi ungekho naye?**		1 = Yes	0 = No<br>**➔SKIP to M33**
M31<br>4.19	Where do you store the expressed breastmilk?<br>**Ulibeka kuphi lolubisisi lwebele olukhanyiwe?**		1= Fridge and/or freezer	2= Elsewhere
M32<br>4.20	What does the carer use to feed the expressed breast milk to the baby?<br>**Kungabe umuntu ogada umntwana usebenzisani ukupha umntwana ubisi lwebele olukhanyiwe?**		1 = Cup / **inkomishi**	2 = Bottle/ **ibhodlela**

M33<br><br>4.21	Do you receive a child support grant for this baby (R350.00)?<br>**Uyayithola i-child support grant ngalomntwana (R350.00)?**	1 = Yes	0 = No

**SECTION 5: HOUSEHOLD INFORMATION (HH)**				
Now I would like to ask for information about the household where the baby is living in.<br>**Ngicela ukukubuza imibuzo mayelana nomuzi ohlala kuwona.**				
**H5**<br><br>5.1	What is the MAIN source of drinking water for this household?<br>**Niwakhaphi amanzi okuphuza ekhaya?**<br><br>***ONLY ONE RESPONSE ALLOWED***<br>***KUVUNYELWE IMPENDULO EYODWA KUPHELA***	1 = Piped – Inside the house<br>***ipayipi ngaphakathi endlini***<br>**➔ *Skip to H9***		
		2 = Piped – Outside but in own yard<br>***ipayipi ngaphendle kwendlu kodwa egcekeni***<br>**➔ *Skip to H9***		
		3 = Piped – outside of the yard<br>***ipayipi- umpompi womphakathi***<br>**➔ *Skip to H8***		
		4 = River water / dam / lake / pond ***amanzi womfula/idamu/ixhaphozi***		
		5 = Tank water (Jojo) / rain water<br>***Amanzi ethanki (ujojo)/ amanzi emvula***		
		6 = Borehole<br>***amanzi aphuma emgodini***		
		7 = Spring surface water<br>***isiphethu***		
		8 = Tanker truck<br>***Imoto yamanzi***		
**H6**<br><br>5.2	Do you do anything to the water to make it safer to drink?<br>**Kukhona enikwenzayo kulamanzi ngaphambi kokuthi niwaphuze, ukwenzela ukuwaphephisa?**	1 = Yes	0 = No<br><br>**➔ *Skip to H8***	-1 = Don't know<br>**➔ *Skip to H8***
H7<br><br>5.3	What do you do to make it safer to drink?<br>**Niwenzani lamanzi ngaphambi kokuthi niwaphuze?**<br><br>***MORE THAN ONE RESPONSE IS ALLOWED***<br>**Impendulo engaphezu kweyodwa ivumelekile**	1 = Boil<br>***niyawabilisa***		
		2 = Add bleach / chlorine<br>***nifaka ujikhi***		
		3 = Strain through a cloth<br>***niyawacwenga ngendwangu***		
		4 = Use water filter / ceramic / sand / composite<br>***sisebenzisa isisefo seceramic/senhlabathi/usebenzisa izinto ezahlukahlukene***		
		5 = Solar disinfectant / leave in the sun<br>***siwagcina elangeni***		
		6 = Let it stand and settle<br>***siyawayeka azike***		
H8<br><br>5.4	How long does it take you to fetch water (there and back)?<br>**Kuthatha isikhathi esingakanani ukukha amanzi (ukuya nokubuya)**	1 = Less than 30 minutes		
		2 = 30 min to 1 hour		
		3 = More than 1 hour		

H9<br><br>5.5	What type of toilet is used by the household?<br><br>**Nisebenzisa hlobo luni lwendlu yangasese?**<br><br>***ONLY ONE RESPONSE ALLOWED KUVUNYELWE IMPENDULO EYODWA KUPHELA***	1 = Flush toilet inside<br>***indlu yangasese esendlini eshawayo***	
		2 = Flush toilet outside<br>***indlu yangasese engaphandle eshawayo***	
		3 = Ventilated pit latrine<br>***indlu yangasese enomgodi nepayipi lomoya***	
		4 = Pit latrine<br>***indlu yangasese enomgodi***	
		5 = Bucket toilet<br>***indlu yangasese esebenzisa ibhakede***	
		6 = Bush / veld / no toilet<br>***ihlathi/ayikho indlu yangasese***	
H10<br><br>5.6	Do you share the toilet with other households?<br>**Ikhona eminye imizi enisebenzisa nayo indlu yangasese?**	1 = Yes	0 = No<br>➔ ***Skip to H13***
H11<br><br>5.7	How many other households share the toilet with you?<br>**Mingaki imizi enisebenzisa nayo indlu yangasese?**	…………….. households	
H13<br>5.8	Is the household connected to electricity?<br>**Umuzi unawo ugesi?**	1 = Yes	0 = No
H14<br><br>5.9	What is the MAIN source of fuel used for cooking food?<br>**Nibasa ngani uma nipheka?**<br><br>***ONLY ONE RESPONSE ALLOWED KUVUNYELWE IMPENDULO EYODWA KUPHELA***	1 = Electricity<br>***ugesi*** ➔ ***Skip to H16***	
		2 = Gas<br>***igesi***	
		3 = Coal<br>***amalahle***	
		4 = Wood<br>***izinkuni***	
		5 = Cow dung<br>***ubulongwe***	
		6 = Paraffin<br>***upharafini***	
		7 = Other<br>***okunye***	
H15<br><br>5.10	Where do you usually cook your food?<br>**Nijwayele ukuphekelaphi ukudla?**	1 = Inside the house	
		2 = Outside the house	
H16<br><br>5.11	CHECK QUESTION: is this the mother of the baby?	1= yes	0 = no<br>➔***Skip to IF11***

SECTION 6: INFANT FEEDING (IF)

**Only mothers are asked this section – if NOT the mother *Skip* to IF11**<br>Now I would to ask you about how you are feeding (name).<br>**Ngithanda ukukubuza mayelana nokudla okunikwa umntwana.**			
IF1	Has (name) ever been breastfed?<br>**U (gama) wake wanceliswa ibele?**	1 = Yes<br>***➔ Skip to IF3***	0 = No
IF2	What was the **main** reason why you decided not to breastfeed?<br>**Yini eyenza ukhethe ukungancelisi u (gama) ibele?**<br><br>***ONLY ONE RESPONSE ALLOWED: ask mother to decide which was most important***	1=unable to establish breastfeeding<br>***angikwazanga ukuncelisa ibele***<br>***➔ Skip to IF11***	
		2 = experiences with/ or perceptions about breastfeeding relating to BABY (e.g. not enough milk/baby crying/baby hungry)<br>***isipiliyoni onaso noma imibono onayo mayelana ngokuncelisa- umntwana (kufaka ubusi olunganele emabeleni/ umntwana ubekhala/ umntwana ubelambile***<br>***➔ Skip to IF11***	
		3 = experiences with/ or perceptions about breastfeeding relating to the MOTHER (e.g. dislike breastfeeding, past bad experience of breastfeeding (painful breasts)<br>***isipiliyoni onaso noma imibono onayo mayelana ngokuncelisa- ngomama (awukuthandanga kuncelisa, unesipiliyoni esembi ngokuncelisa(amabele abuhlungu)***<br>***➔ Skip to IF11***	
		4= had to go back to work or school (or be away from the baby)<br>***kwakumele ngibuyele emsebenzini noma esikoleni***<br>***➔ Skip to IF11***	
		5 = mother's health (including HIV)<br>***isimo sempilo sikamama, singabala nesandulela ngculazi***<br>***➔Skip to IF11***	
		6 = A health worker advised me not to breastfeed<br>***umsebenzi wezempilo wangeluleka ukuba nginga ncelisi***<br>***➔ Skip to IF11***	
		7 = My mother/partner/family member advised me not to breastfeed<br>***umama wami/umuntu engizwana naye/amalunga omndeni angiluleka ukuba ngingancelisi***<br>***➔ Skip to IF11***	
		8 = Other or no response<br>***okunye noma akaphendulanga***<br>***➔ Skip to IF11***	
IF3	Are you still breastfeeding (name)?<br>**Usamncelisa ibele u (gama)?**	1 = Yes<br><br>***➔ Skip to IF8***	0 = No

IF4	For how long did you breastfeed (name)?<br>**Wamncelisa isikhathi esingakanani u (gama)?**	**FOR 14 WEEK AGE GROUP**<br>1 = <1 week
		2 = 1-2 weeks
		3 = 3-4 weeks
		4 = 5-6 weeks
		5 = 7-8 weeks
		6 = 9-10 weeks
		7 = 11-12 weeks
		8 = 13-14 weeks
		**FOR 6 MONTH AGE GROUP**<br>9 = less than one month
		10= 1- <2 months
		11= 2- <3 months
		12=3 - < 4 months
		13 = 4= < 5months
		14= 5- 6months
IF5	What is the **main** reason why you decided to stop breastfeeding (name)?<br>**Wayekelani ukuncelisa u (gama) ibele?**<br><br>***ONLY ONE RESPONSE ALLOWED: ask mother to decide which was most important***	2 = experiences with/ or perceptions about breastfeeding - BABY (includes not enough milk/baby crying/previous bad experience)<br>***Isipiliyoni onaso noma imibono onayo mayelana ngokuncelisa- umntwana (kufaka ubusi olunganele emabeleni, umntwana ubekhala, akukuphathanga kahle ukuncelisa phambilini)***<br>➔ ***Skip to IF9***
		3 = experiences with/ or perceptions about breastfeeding – MOTHER e.g. dislike breastfeeding, past bad experience of breastfeeding (painful breasts)<br>***Isipiliyoni onaso noma imibono onayo mayelana ngokuncelisa- ngomama (awukuthandanga kuncelisa, unesipiliyoni esembi ngokuncelisa)amabele abuhlungu***<br>➔ ***Skip to IF9***
		4= had to go back to work or school (or be away from the baby)<br>***kwakumele ngibuyele emsebenzini noma esikoleni***<br>➔ ***Skip to IF9***
		5 = mother's health incuding HIV<br>***isimo sempilo sikamama, singabala nesandulela ngculazi***<br>➔ ***Skip to IF9***
		6 = A health worker advised me not to breastfeed<br>***umsebenzi wezempilo wangeluleka ukuba nginga ncelisi***<br>➔ ***Skip to IF9***
		7 = My mother/partner/family member advised not to breastfeed<br>***umama wami/umuntu engizwana naye/amalunga omndeni angiluleka ukuba ngingancelisi***<br>➔ ***Skip to IF9***
		8 = Other or no response<br>***(okunye noma akaphendulanga)*** ➔ ***Skip to IF9***

IF8	Has (name) ever been given any other foods or fluids other than breast milk?<br>**U (gama) wake wanikwa okunye ukudla noma okusaketshezi ngaphandle kobisi lwebele?**	1 = Yes	0 = No<br><br>**➔SKIP to IF11**
IF9	At what age did you start to give (name) other foods or fluids other than breastmilk?<br>**Waqala nini ukunika u (gama) okunye ukudla noma okusaketshezi?**	**FOR 14WEEK AGE GROUP**<br>1 = (<1 week old)	
		2 = (1-2 weeks old)	
		3 = (3-4 weeks old)	
		4 = (5-6 weeks old)	
		5 = (7-8 weeks old)	
		6 = (9-10 weeks old)	
		7 = (11-12 weeks old)	
		8 = (13-14 weeks old)	
		**FOR SIX MONTH AGE GROUP**<br>9 = less than one month	
		10 = 1- <2months	
		11 = 2 - < 3months	
		12 = 3- <4months	
		13= 4- <5months	
		14= 5-6months	
IF10	What was the **main** reason why you started to give your baby other food or fluids?<br>**Yini eyakwenza waqala ukunika u (gama) okunye ukudla noma okusaketshezi?**<br><br>**DO NOT PROMPT**<br><br>**More than one response allowed: mark any options that applies**	2 = experiences with/ or perceptions about breastfeeding - BABY (includes not enough milk/baby crying/previous bad experience)<br>***Isipiliyoni onaso noma imibono onayo mayelana ngokuncelisa- umntwana (kufaka ubusi olunganele emabeleni, umntwana ubekhala, akukuphathanga kahle ukuncelisa phambilini)***	
		3 = experiences with/ or perceptions about breastfeeding – MOTHER e.g. dislike breastfeeding, past bad experience of breastfeeding (painful breasts)<br>***Isipiliyoni onaso noma imibono onayo mayelana ngokuncelisa- ngomama (awukuthandanga kuncelisa, unesipiliyoni esembi ngokuncelisa) amabele abuhlungu***	
		4= had to go back to work or school (or be away from the baby)<br>***kwakumele ngibuyele emsebenzini noma esikoleni***	
		5 = mother's health including HIV<br>***isimo sempilo sikamama, singabala nesandulela ngculazi***	
		6 = A health worker advised me not to breastfeed<br>***umsebenzi wempilo wangeluleka ukuba nginga ncelisi***	
		7 = My mother/partner/family member advised not to breastfeed<br>***umama wami/umuntu engizwana naye/amalunga omndeni angiluleka ukuba ngingancelisi***	
		8 = Other or no response<br>***okunye noma akaphenduli***	

IF10_1	What was the first food or fluid that you gave the baby in addition to breastmilk?<br>**Ikuphi ukudla noma okusaketshezi okwaba okokuqala owakupha umntwana ngesikhathi usunezela obisini lwebele?**	1=Water<br>***amanzi***	
		2= Infant formula milk<br>***ubisilwebhodlela***	
		3=Condensed or evaporated milk, powdered or fresh animal milk<br>***ubisi lwempuphu noma ubisi lwesilwane (inkomo)***	
		4=Yoghurt, amasi or thin porridge<br>***iyogathi, amasi, iphalishi***	
		5=Traditional medicine<br>***umuthi wesintu***	
		6=Other<br>***okunye***	
IF10_2	What was the second food or fluid you gave the baby in addition to breastmilk?<br>**Ikuphi ukudla noma okusaketshezi okwaba okwesibili owakupha umntwana ngesikhathi usunezela obisini lwebele?**	1=Water<br>***amanzi***	
		2=Infant formula milk<br>***ubisilwebhodlela***	
		3=condensed or evaporated milk, powdered or fresh animal milk<br>***ubisi lwempuphu noma ubisi lwesilwane (inkomo)***	
		4=Yoghurt, amasi or thin porridge<br>***iyogathi, amasi, iphalishi***	
		5=Traditional medicine<br>***umuthi wesintu***	
		6=Other<br>***okunye***	
IF11	Are you able to report about how (name) has been fed for the last 24 hours?<br>**Ungakwazi ukungitshela ukuthi u(gama) utholeni okudliwayo emahoreni awu 24 adlule kusukela ngalesikhathi izolo?**	1 = Yes	0 = No ➔ ***Skip to K1***

TO BE ASKED FROM BOTH MOTHERS AND NON-MOTHER RESPONDENTS

I would like to ask you particularly about any liquids and semi-solid / solid food (with a spoon) that (name) may have had ***yesterday during the day and night (24 hours).*** I am interested in whether (name) had the item even if it was combined with other food.
I will start with liquids.
Ngicela ukukubuza ngokusaketshezi noma ukudla okuthambile okudliwe umntwana izolo emini noma ebusuku?
Ngizoqala ngokungamanzi.

IF12	Did (name) drink plain water yesterday during the day or night?<br>**Ngabe u(gama) uke waphuza amanzi engaxutshwe nalutho emini noma ebusuku?**	1 = Yes	0 = No
IF13	Did (name) drink infant formula yesterday during the day or night?<br>**Ngabe u(gama) uke waphuza ifomula izolo emini noma ebusuku?**	1 = Yes	0 = No<br>***Skip to IF15***
IF14	How many times did (name) drink infant formula during the day and night?<br>**Ngabe u(gama) uyiphuze kangaki ifomula izolo emini noma ebusuku?**	………………………….. times	

IF15	Did (name) drink tinned milk (Condensed milk or evaporated Milk), powdered milk or fresh animal (cows) milk yesterday during the day or night?<br>**Ngabe u(gama) uke waphuza ubisi lwethini (ikhondense), olwempuphu noma ubisi lwesilwane olu-fresh izolo emini noma ebusuku?**	1 = Yes	0 = No<br>***Skip to IF17***
IF16	How many times did (name) drink tinned milk, powdered milk or fresh animal milk during the day and night?<br>**Ngabe u(gama) uliphuze kangaki ubisi lwethini, olwempuphu noma ubisi lwesilwane olu-fresh izolo emini noma ebusuku?**	………………………… times	
IF17	Did (name) drink juice or juice drinks or tea yesterday during the day or night?<br>**Ngabe u(gama) uke waphuza ijusi noma itiye izolo emini noma ebusuku?**	1 = Yes	0 = No
IF18	Did (name) drink clear broth/clear soup yesterday during the day or night?<br>**Ngabe u(gama) uke waphuza isobho izolo emini noma ebusuku?**	1 = Yes	0 = No
IF19	Did (name) drink or eat vitamin or mineral supplements or ORS or any medicines *obtained from the clinic* or a doctor yesterday during the day or night?<br>**Ngabe u(gama) uke waphuza ama-vitamin, nama-minerals noma i-ORS noma imithi oyithole emtholampilo noma kudokotela izolo emini noma ebusuku?**	1 = Yes	0 = No
IF20	Did (name) drink or eat any muthi e.g. muthi nyoni,or traditional medicines *obtained from a traditional healer*, or bought over the counter yesterday during the day or night?<br>**Ngabe u(gama) uke waphuza noma wadla imithi yesintu etholwe kumlaphi wendabuko noma othengwe ekhemisti izolo emini noma ebusuku?**	1 = Yes	0 = No
IF21	Did (name) drink any other liquids yesterday during the day or night that I have not mentioned?<br>**Kukhona okunye okusaketshezi akuphuzile u(gama) izolo emini noma ebusuku?**	1 = Yes	0 = No
IF22	Did (name) drink or eat yogurt or amaasi or thin porridge yesterday during the day or night?<br>**Ngabe u(gama) uke waphuza noma wadla iyogathi, amaasi noma iphalishi elimanzi izolo emini noma ebusuku?**	1 = Yes	0 = No
Now I will ask you about solid or semi-solid mushy food.<br>**Manje ngizokubuza ngokudla okuqinile noma okuthambile.**			
IF24	Did (name) eat solid or semi-solid food (mushy soft food) yesterday during the day or night?<br>**Ngabe u(gama) uke wadla ukudla okuthambile noma okuqinile izolo emini noma ebusuku?**	1 = Yes	0 = No<br>***Skip to IF26***
IF25	How many times did (name) eat solid or semi-solid food (mushy soft food) yesterday during the day or night?<br>**Ukudle kangaki u(gama) ukudla okuthambile noma okuqinile izolo emini noma ebusuku?**	…………………. times	
IF26	Did (name) have any commercial baby cereal (cerelac) yesterday during the day or nights?<br>**Ngabe u(gama) uke wadla ipharishi elithengwe esitolo (cerelac) izolo emini noma ebusuku?**	1 = Yes	0 = No

IF27	Did (name) have bread, rice, porridge or other foods made from grains yesterday during the day or night?<br>**Ngabe u(gama) uke wadla isinkwa, i-rice, i-pharishi noma okunye ukudla okwenziwe ngokolweni izolo emini noma ebusuku?**	1 = Yes	0 = No
IF28	Did (name) have any fruits or vegetables yesterday during the day or night?<br>**Ngabe u(gama) uke wadla izithelo izolo emini noma ebusuku?**	1 = Yes	0 = No
IF29	Did (name) have meat (beef, pork, lamb) or poultry (chicken) yesterday during the day or night?<br>**Ngabe u(gama) uke wadla inyama (ebovu, eyengulube, eyemvu) noma eyenkukhu izolo emini noma ebusuku?**	1 = Yes	0 = No
IF30	Did (name) have egg yesterday during the day or night?<br>**Ngabe u(gama) uke wadla iqanda izolo emini noma ebusuku?**	1 = Yes	0 = No
IF31	Did (name) have any fish or seafood (fresh fish, canned fish, sardines, pilchards, tuna,) yesterday during the day or night?<br>**Ngabe u(gama) uke wadla u-fishi izolo emini noma ebusuku?**	1 = Yes	0 = No
IF32	Did (name) have any cheese or other foods made from milk (custard etc.) yesterday during the day or night?<br>**Ngabe u(gama) uke wadla u-cheese noma okunye ukudla okwenziwe ngo bisi (custard) izolo emini noma ebusuku?**	1 = Yes	0 = No
IF33	Did (name) have any sugar and sweets (chocolates, cake, biscuits, honey, sweetened drinks sugary foods,) yesterday during the day or night?<br>**Ngabe u(gama) uke wadla ushukela noma uswidi (amakhekhe, uju, ukudla okunoshukela) izolo emini noma ebusuku?**	1 = Yes	0 = No
IF34	Did (name) have any other solid or semi-solid food that I have not mentioned yesterday during the day or night?<br>**Ngabe u(gama) uke wadla ukudla okuqinile noma okuthambile engingakubalanga izolo emini noma ebusuku?**	1 = Yes	0 = No
IF35	CHECK QUESTION: Is this the mother of the child?<br>**Ingabe ungumama womntwana**	1 = Yes	0 = No ➔ ***Skip to R1***

SECTION 7: BREASTFEEDING KNOWLEDGE AND ATTITUDES (K)

Now I would like to you about issues regarding breastfeeding and what you think. I will read out some statements to you and you must please tell me if you think the statement is true or false.<br>**Ngicela ukubuza mayelana nokucabangayo ngokuncelisa. Ngizokubuza wena ungitshele ukuthi iqiniso noma cha.**			
K1	Initial breast production of yellow water (colostrum) is nutritionally useless for the baby and should be discarded<br>**Ubisi lokuqala oluphuma ebeleni (colostrum) alumsizi umntwana futhi aludingeki.**	1=True	0= False
K2	Three months of breastfeeding is long enough<br>**Izinyanga ezintathu zoku ncelisa umntwana ibele zanele.**	1=True	0= False
K3	Breastfed babies have less diarrhoea<br>**Abantwana abancela ibele ababi nesifo sohudo kakhulu**.	1=True	0= False
K4	Infant formula contains all the ingredients found in human breastmilk<br>**Ubisi lwebhodlela lunezithako zonke ozithola ebeleni lamama.**	1=True	0= False
K5	Breastfeeding is easier than feeding with infant formula<br>**Ukuncelisa ibele kulula kunokuncelisa umntwana ubisi lwebhodlela.**	1=True	0= False
K6	Doctors and nurses encourage breastfeeding<br>**oDokotela kanye no-nesi bayakugqugquzela ukuncelisa umntwana ibele.**	1=True	0= False
K7	A mother who feels that the baby is not getting enough breastmilk should top up the feeds with formula milk<br>**Uma umama ezwa ukuthi ukuncelisa ibele akumnelisi umntwana kumele anezezele ngobisi lwebhodlela.**	1=True	0= False
K8	Most women in my community have breastfed their babies<br>**Iningi labantu besifazane emphakathini wakho bebencelisa abantwana babo ubisi lwebele.**	1=True	0= False
K9	I am worried that my breasts will lose their shape if I breastfeed my baby<br>**Ukhathazwa ukuthi amabele akho azo shintsha isimo sawo uma uncelisa ubisi lwebele**	1=True	0= False
K10	My community encourages breastfeeding over feeding with infant formula<br>**Umphakathi wakho uyakugqugquzela ukuncelisa umntwana ibele kunokuncelisa ifomula.**	1=True	0= False
K11	I feel comfortable to breastfeed my baby wherever I am<br>**Ukhululekile ukuncelisa umntwana ibele noma ikuphi la ukhona.**	1=True	0= False
K12	I would be willing to donate (give) my breastmilk to a sick baby if the mother did not have enough milk<br>**Ngeke ubenenkinga ngokunikela ngobisi lwakho lwebele emntwaneni ogulayo uma umama waloyomntwana engenalo ubisi olwanele.**	1=True	0=False
K13	How old should the baby be when you start giving other food and fluids?<br>**Kumele umntwana abengakanani kuze uqale ukumnika okunye ukudla?**	1= 6 months	2= Other

K14	It is better to give even a small amount of breastmilk to the baby rather than giving no breastmilk at all<br>**Kungcono ukunika umntwana ubisi oluncane lwebele kunokuthi ungamuniki nhlobo ubisi lwebele**	1=True	0=False
K15	Expressed breastmilk can be stored outside of the fridge for upto 12 hours<br>**Ubisi lwebele olukhanyiwe lingakwazi ukuhlala ngaphandle kwe-fridge amahhora ayishumi nambil**i	1=True	0=False
K16	Once the baby starts taking other food or fluids it is better to stop breastfeeding completely<br>**Uma umntwana waqala wadla okunye ukudla noma okusaketshezi kungcono ukuthi uyeke ukuncelisa ibele?**	1=True	0=False
K17	If you want to express breastmilk to feed your baby while you are away for him/her it is best to express breastmilk using a machine that you can buy in the shop<br>**Kungcono ukukhama ubisi lwebele ngomshini othengwa esitolo**	1=True	0=False

SECTION 8: EXPOSURE TO BREASTFEEDING COUNSELLING OR ADVICE (C).

I would like to ask you information about your pregnancy, your delivery and where or when you got advice about how to feed your baby. Firstly, I am going to ask you about the feeding advice you received from health workers during your antenatal visits to the clinic

Ngicela ukubuza mayelana ngokukhulelwa kwakho, ukuteta, nokuthi wazitholaphi izeluleko zokuncelisa umntwana. Okokuqala, ngizokubuza ngezeluleko ngokuncelisa kanye nezinye izeluleko owazithola kunompilo ngesikhathi uyoxukuza ekliniki.

C1	Did you attend the clinic for antenatal care during this last pregnancy?<br>**Waya emtholampilo ukuyoxukuza ngesikhathi ukhulelwe umagcino wakho?**	1 = Yes	0 = No<br>➔ ***Skip to C4***
C2	When you attended the ANC, did a health worker give you advice about how you were going to feed your baby while you were still pregnant?<br>**Ngesikhathi uyoxukuza ukhona owezempilo owakunika izeluleko mayelana nendlela okumele uphe ngayo umntwana wakho ukudla?**	1 = Yes	0 = No<br>➔ ***Skip to C4***
C2_1	During the ANC did anyone advise you about expressing breastmilk to feed your baby when you are away from him/her?<br>**Ngesikhathi uyoxukuza kukhona yini owakunika izeluleko zokuthi ungalukhama kanjani ubisi lwebele ukuze uphe umntwana ngesikhathi ungekho naye?**	1 = Yes	0 = No<br>➔ ***Skip to C4***
C3	Who gave you advice about how you were going to feed your baby while you were still pregnant?<br>**Ubani owakunika izeluleko mayelana nendlela ozonika ngayo umntwana wakho ukudla ngalesiskhathi ukhulelwe?**	1 = Nurse<br>2 = HIV counsellor<br>3 = Nutritional Advisor<br>4 = CCG in the clinic<br>5 = Other/ don't know category of health worker	

C4	Has a CCG or community nurse ever visited you in your home?<br>**Uke wavakashelwa unompilo noma unesi womphakathi ekhaya?**	1 = Yes	0 = No ➔ ***Skip to C7***
C5	Did a CCG or community nurse visit you while you were pregnant in your home?<br>**Wakuvakashela unompilo noma unesi womphakathi ngesikhathi ukhulelwe?**	1 = Yes	0 = No ➔ ***Skip to C7***
C6	Did a CCG or community nurse give you advice on how you would feed your baby while you were pregnant?<br>**Ngesikhathi ukhulelwe unompilo wakhuluma nawe mayelana nokuthi kufanele umntwana umncelise kanjani ngemuva kokuzalwa?**	1 = Yes	0 = No

Now I am going to ask you about the feeding advice you received from health workers at the facility when (name) was born.
Manje ngizokubuza mayelana nezeluleko owazithola konompilo mayelana nendlela yokunika umntwana wakho ukudla esikhungweni sezempilo la owathola umntwana wakho.

C7	Where was (name) born?<br>**Wazalelwa kuphi umntwana?**	1 = Clinic<br>2= Hospital<br>3 = Outside a health facility but went into health facility afterwards<br>4= Outside a health facility and never went into health facility ➔ ***Skip to C13***	
C7_1	How was (name) delivered?<br>**Ingabe wamutete kanjani u (gama)?**	1=Vaginal delivery	2=Caesarean section
C8	Did anyone at the health facility where (name) was born give you advice on how to feed your baby?<br>**Ingabe ukhona yini owakululeka ngokupha ingane ukudla esikhungweni sezempilo lapho kwazalakhona u(gama)?**	1 = Yes	0 = No<br><br>➔ ***Skip to C10***
C9	Who advised you about how to feed (name) when you were in the health facility when (name) was born?<br>**Ubani owakunika izeluleko mayelana nendlela yokunika umntwana wakho ukudla ngesikhathi usesikhungweni sezempilo ngesikhathi eyobelethe umntwana wakho?**	1 = Nurse<br>2 = Community Care Giver<br>3 = Lay counsellor<br>4 = Lactation advisor<br>5 = Other/don't know category of health worker	
C10	Straight after your baby was born was your baby placed on your tummy/chest?<br>**Umntwana wakho wabekwa esiswini noma esifubeni sakho ngemuva kokuzalwa (ngaphambi kokuthi kusikwe inkaba)?**	1 = Yes	0 = No<br><br>➔ ***Skip to C13***
C11	When the baby was placed on your tummy/chest was your baby placed skin-to-skin?<br>**Ngesikhathi umntwana ebekwa esiswini noma esifubeni sakho, kwakuyinyama enyameni?**	1 = Yes	0 = No
C13	In the first few days after (name) was born, did you give (name) any foods or fluids (other than breast milk) while you were waiting for your milk?<br>**Ezinsukwini ezimbalwa umntwana ezelwe wamnika okunye ukudla noma okungamanzi ngesikhathi ulinde ubisi luphume emabeleni**	1 = Yes	0 = No<br><br>➔***Skip to C14***

<table>
<tr><td rowspan="4">C13_1</td><td colspan="2" rowspan="4">What food or fluid did you give while you were waiting for your milk?
Yiluphi uhlobo lokudla noma isiphuzo owasipha umntwana ngesikhathi ulinde ubisi lwakho ukuthi lungene?
MORE THAN ONE OPTION ALLOWED</td><td colspan="2">1 = Water</td></tr>
<tr><td colspan="2">2 = Formula milk</td></tr>
<tr><td colspan="2">3 = Breast milk</td></tr>
<tr><td colspan="2">4 = Other</td></tr>
<tr><td rowspan="5">C14</td><td colspan="2" rowspan="5">When (name) was born, what was the very first fluid that he/she received?
Ikuphi okokuqala okusaketshezi okwatholwa umntwana esanda kuzalwa?</td><td colspan="2">1 = Water</td></tr>
<tr><td colspan="2">2 = Formula milk</td></tr>
<tr><td colspan="2">3 = Breast milk</td></tr>
<tr><td colspan="2">4 = Other</td></tr>
<tr><td colspan="2">5 = Someone else gave first feed</td></tr>
<tr><td rowspan="3">C15</td><td colspan="2" rowspan="3">How soon after (name) was born did you first breastfeed your baby?
Kwathatha isikhathi esingakananani emuva kokuba u(gama) esezelwe ukuthi umnike ubisi lwebele?</td><td colspan="2">1 = Within 1 hour of delivery</td></tr>
<tr><td colspan="2">2 = After 1 hour from delivery</td></tr>
<tr><td colspan="2">-1 = Do not know</td></tr>
<tr><td rowspan="3">C16</td><td rowspan="3">Where did you first breastfeed (name)?
Wamuncelisela kuphi okokuqala u(gama)?</td><td colspan="3">1 = While you were still in the delivery room
ngesikhathi ngisasegumbini lokuzala</td></tr>
<tr><td colspan="3">2 = When you had moved to the postnatal ward
ngesikhathi sengi sengidluliselwe egunjini labantu abazele</td></tr>
<tr><td colspan="3">3= other
okunye</td></tr>
<tr><td>C17</td><td colspan="2">Did anyone help you with breastfeeding after (name) was born?
Ukhona owakusiza ngokuncelisa u(gama) ibele esezelwe?</td><td>1 = Yes</td><td>0 = No
➔ Skip to C19</td></tr>
<tr><td rowspan="5">C18</td><td rowspan="5">Who helped you with breastfeeding after (name) was born?
Ubani owakusiza ngokuncelisa ibele ngemuva kokuzala u(gama)?</td><td colspan="3">1 = Nurse</td></tr>
<tr><td colspan="3">2 = Community Care Giver</td></tr>
<tr><td colspan="3">3 = Lay counsellor or peer counsellor</td></tr>
<tr><td colspan="3">4 = Lactation (Breastfeeding) advisor</td></tr>
<tr><td colspan="3">5 = Other/don't know category of health worker</td></tr>
<tr><td>C19</td><td colspan="2">Were you seen by a lactation advisor after delivery?
Ukhona umluleki wezokuncelisa owakubona ngemuva kokubeletha?</td><td>1 = Yes</td><td>0 = No ➔ Skip to C20_1</td></tr>
<tr><td>C20</td><td colspan="2">How many times were you seen by a lactation (breastfeeding) advisor?
Wakubona kangaki umluleki wezokuncelisa?</td><td colspan="2">…………………….. times</td></tr>
<tr><td>C20_1</td><td colspan="2">While you were in health facility did anyone advise you about expressing breastmilk to feed the baby when you are away from him/her?
Ngesikhathi usesikhungweni sezempilo ukhona owakweluleka ngokukhama ubisi lwebele lokupha umntwana ngesikhathi ungekho naye?</td><td>1 = Yes</td><td>0 = No</td></tr>
</table>

Now I would like to ask you about what happened when you went home after (name) was born.<br>**Manje ngithanda ukubuza ngokwenzeka ekhaya emva kokuteta?**			
C21	Did a CCG or community nurse visit you in your home since (name) was born?<br>**Ukhona unompilo noma unesi womphakathi okuvakashele ekhaya ngemuva kokuzalwa kuka (gama)?**	1 = Yes	0 = No ➔ ***Skip to C24***
C22	How many times has a CCG or community nurse visited you in your home since (name) was born?<br>**Ngabe usuke wavakashelwa kangaki unompilo noma unesi womphakathi ekhaya kusukela kuzelwe u(gama)?**	……………………… times	
C23	Did the CCG or community nurse advise you about how to feed (name)?<br>**Ngabe unompilo noma unesi womphakathi uke wakucebisa noma akululeke ngendlela ekufanele unike ngayo u(gama) ukudla?**	1 = Yes	0 = No
C23_1	Did a CCG or community nurse advise you about how to express breastmilk to feed your baby when you are away from him/her?<br>**Ngabe unompilo noma unesi womphakathi uke wakucebisa noma akululeke ngokusenga ubisi lwebele ukuze adle umntwane uma ungekho eduze kwakhe?**	1 = Yes	0 = No
C24	Have you been advised about feeding your baby by a community or family member?<br>**Ngabe likhona ilunga lomphakathi noma elomndeni elakunikeza izeluleko mayelana nendlela ekufanele unike ngayo umntwana ukudla?**	1 = Yes	0 = No ➔ ***Skip to FF1***
C25	Who advised you about feeding your baby?<br>**Ubani umuntu owakunika izeluleko mayelana ngendlela ekufanele unike ngayo umntwana wakho ukudla?**<br><br>More than one response allowed: mark any options that apply	1 = Family member<br>2 = Non family member<br>3= Traditional health practitioner<br>4= other	

SECTION 9: FORMULA FEEDING (FF)			
FF1	Have you ever given (name) any formula milk?<br>**Wake wancelisa u(gama) ifomula?**	1 = Yes	0 = No<br>➔ ***Skip to FF4***
FF1_2	How old was (name) when you first gave him formula milk?<br>**Wayengakanani u(gama) ngesikhathi uqala ukumncelisa ifomula?**	**FOR 14WEEK AGE GROUP**<br>1 = (<1 week old)<br>2 = (1-2 weeks old)<br>3 = (3-4 weeks old)<br>4 = (5-6 weeks old)<br>5 = (7-8 weeks old)<br>6 = (9-10 weeks old)<br>7 = (11-12 weeks old)<br>8 = (13-14 weeks old)	

		**FOR SIX MONTH AGE GROUP**<br>9 = less than one month<br>10 = 1- <2months<br>11 = 2 - < 3months<br>12 = 3- <4months<br>13= 4- <5months<br>14= 5-6months	
FF2	Who advised you to give formula milk to (name)?<br>**Ubani owakweluleka ngokunika u(gama) ifomula?**<br><br>**More than one response is allowed**	1 = Decided myself that I wanted to formula feed<br>2 = Professional health worker / nurse<br>3 = Non-professional health worker e.g. CCG / Lay health counsellor<br>4 = Family member e.g. grandmother<br>5 = Non family member e.g. neighbour / community member/traditional healer	
FF3	Has a health worker given you advice about how to prepare the feeds (mix the feed, sterilise the bottles etc)?<br>**Ukhona umsebenzi wezempilo oke wakweluleka ngendlela yokulungisela umntwana wakho ubisi lwebhodlela? (ukuxuba ubisi, noku bilisa amabhodlela)**	1 = Yes	0 = No
FF4	Have you ever given (name) plain water?<br>**Wake wamupha u (gama) amanzi angaxutshwe naluthi?**	1 = Yes	0 = No<br>**SKIP to FF6**
FF5	How old was (name) when you first gave water?<br>**Waye neminyaka emingaki u (gama) ngesikhathi umupha amanzi okokuqala?**	**FOR 14WEEK AGE GROUP**<br>1 = (<1 week old)<br>2 = (1-2 weeks old)<br>3 = (3-4 weeks old)<br>4 = (5-6 weeks old)<br>5 = (7-8 weeks old)<br>6 = (9-10 weeks old)<br>7 = (11-12 weeks old)<br>8 = (13-14 weeks old)<br>**FOR SIX MONTH AGE GROUP**<br>9 = less than one month<br>10= 1- <2months<br>11 = 2 - < 3months<br>12 = 3- <4months<br>13= 4- <5months<br>14= 5-6months	
FF6	Have you ever given (name) traditional medicines or muthi from a traditional healer?<br>**Wake wayipha ingane imithi yesintu noma umuthi oqhamuka kum'laphi wesintu?**	1 = Yes	0 = No<br><br>➔ **SKIP to HIV1**
FF7	How old was the baby when you first gave him traditional medicine? **Wayeneminyaka emngaki u (gama) umunika umuthi wesintu okokuqala?**	FOR 14WEEK AGE GROUP<br>1 = (<1 week old)<br>2 = (1-2 weeks old)<br>3 = (3-4 weeks old)<br>4 = (5-6 weeks old)<br>5 = (7-8 weeks old)	

		6 = (9-10 weeks old)
		7 = (11-12 weeks old)
		8 = (13-14 weeks old)
		FOR SIX MONTH AGE GROUP<br>9 = less than one month
		10= 1- < 2months
		11 = 2 - < 3months
		12 = 3- <4months
		13= 4- <5months
		14= 5-6months

SECTION 10: HIV (HIV)

I am going to ask you questions about HIV. Please remember that everything you tell me is confidential and that you do not have to answer any question that you do not choose to answer. When I ask the question, please just tell me that you prefer not to answer that question. This will not in any way impact on the care you receive at this facility.

Ngizokubuza imibuzo ethize maqondana neGCIWANE LESANDLULELA NGCULAZI (iHIV). Siza ukhumbule ukuthi konke ongitshela kona kuyimfihlo futhi AKUMELE uphendule noma yimuphi umbuzo ongaFISI ukuwuphendula. Uma ngikubuza umbuzo, siza ungitshele ukuthi AWUTHANDI ukuwuphendula lowo mbuzo. Lokhu ngeke KUTHUNAZE ukunakekelwa kwakho okuthola kulesi sikhungo.

HIV1	Were you tested for HIV infection during your last pregnancy?<br>**Wahlolelwa igciwane lesandulela ngculaza ekukhulelweni kwakho okokugcina?**	1 = Yes<br>➔ ***Skip to HIV3***	2 = No	-1 = Do not know	-3 = Choose not to answer ➔ ***Skip to R1***
HIV2	Have you ever been tested for HIV?<br>**Usuke walihlolela igciwane lesandulela ngculaza?**	1 = Yes	2 = No<br>➔ ***Skip to R1***	-1 = Do not know<br>➔ ***Skip to R1***	-3 = Choose not to answer ➔ ***Skip to R1***
HIV3	What was the result of your most recent HIV test?<br>**Yayithini imiphumela?**	1 = Infected / HIV-positive	2 = Not infected / HIV-negative<br>➔ ***Skip to HIV10***		-3 = Chooses not to answer<br>➔ ***Skip to R1***
HIV4	Are you taking ARVs or FDC?<br>**Uyawadla ama-ARV's noma i-FDC?**	1 = Yes	2 = No		-3 = chooses not to answer
HIV5	Has the baby had a PCR test?<br>**Wake wayenza i-PCR test umntwana?**	2 = Yes	2 = No<br>➔ ***Skip to HIV8***		-3 = chooses not to answer
HIV6	What was the result of the PCR test?<br>**Yayithini imphumela?**	1= positive	2= negative	-1=Do Not Know	-3 = chooses not to answer
HIV7	Is the baby on antiretroviral treatment?<br>**Ingabe umntwana uyayidla i-antiretroviral treatment?**	1 = Yes	2 = No		-3 = chooses not to answer

HIV8	Have you had blood taken for a viral load count?<br>**Usuke walithatha igazi lokwenza i-viral load count?**	1 = Yes	2 = No<br>➔ ***Skip to R1***	-1 = Do not know<br>➔ ***Skip to R1***
HIV9	When was the last time you had blood taken for a viral load count?<br>***Wagcina nini ukuthatha igazi lokwenza i-viral load count?***	1 = within the last 6 months<br>➔ ***Skip to R1***	2 = longer than 6 months ago<br>➔ ***Skip to R1***	-1 = Do not know<br>➔ ***Skip to R1***
HIV10	When last were you re-tested for HIV?<br>**Wagcina nini ukuhlolela futhi igciwane lesandulela ngculazi?**	1 = Within the last 3 months	2 = Longer than 3 months ago	-1 = Do not know

SECTION 11: ROAD TO HEALTH CARD

Now I would like to ask you if I may look at your Road to Health Card. I would like to look at how your baby is growing.
Manje ngicela ukubuka i-Road to Health Card yakho. Ngithanda ukubheka ukuthi umntwana wakho ukhula kanjani.

R1	Is the Road to Health Card present?<br>**Ingabe likhona i-card le-Road to Health?**	1 = Yes				0 = No<br>➔ ***Skip to R36***			
R2 G	What is the birthweight recorded on the RTHC?	☐☐☐☐ g							
R3	Is there another weight recorded (WEIGHT 2)?<br>Do not include todays weight	1 = Yes				0 = No<br>➔**SKIP to R18**			
R4	What is the date for WEIGHT 2?	D	D	M	M	Y	Y	Y	Y
R5	What is the value of WEIGHT 2?	☐☐☐☐ g							
R6	Is there another weight recorded (WEIGHT3)?<br>Do not include todays weight	1 = Yes				0 = No<br>➔**SKIP to R18**			
R7	What is the date for WEIGHT 3?	D	D	M	M	Y	Y	Y	Y
R8	What is the value of WEIGHT 3?	☐☐☐☐ g							
R9	Is there another weight recorded (WEIGHT 4)?<br>Do not include todays weight	1 = Yes				0 = No<br>➔**SKIP to R18**			
R10	What is the date for WEIGHT 4?	D	D	M	M	Y	Y	Y	Y
R11	What is the value of WEIGHT 4?	☐☐☐☐ g							
R12	Is there another weight recorded (WEIGHT5)?<br>Do not include todays weight	1 = Yes				0 = No<br>➔**SKIP to R18**			

R13	What is the date for WEIGHT 5?	D	D	M	M	Y	Y	Y	Y
R14	What is the value of WEIGHT 5?	☐☐☐☐ g							
R15	Is there another weight recorded (WEIGHT6)?<br>Do not include todays weight	1 = Yes				0 = No<br>➔**SKIP to R18**			
R16	What is the date for WEIGHT 6?	D	D	M	M	Y	Y	Y	Y
R17	What is the value of WEIGHT 6?	☐☐☐☐ g							
R18	What is the weight recorded today?	☐☐☐☐ g							
R19G	What is the birth length recorded on the RTHC?	☐☐☐ cm							
R20	Is there another length recorded (LENGTH 2)?<br>Do not include todays length	1 = Yes				0 = No<br>➔**SKIP to R35**			
R21	What is the date for LENGTH 2?	D	D	M	M	Y	Y	Y	Y
R22	What is the value of LENGTH 2?	☐☐☐ cm							
R23	Is there another weight recorded (LENGTH 3)?<br>Do not include todays length	1 = Yes				0 = No<br>➔**SKIP to R35**			
R24	What is the date for LENGTH 3?	D	D	M	M	Y	Y	Y	Y
R25	What is the value of LENGTH 3?	☐☐☐ cm							
R26	Is there another weight recorded (LENGTH 4)?<br>Do not include todays length	1 = Yes				0 = No<br>➔**SKIP to R35**			
R27	What is the date for LENGTH 4?	D	D	M	M	Y	Y	Y	Y
R28	What is the value of LENGTH 4?	☐☐☐ cm							
R29	Is there another weight recorded (LENGTH 5)?<br>Do not include todays length	1 = Yes				0 = No<br>➔**SKIP to R35**			

R30	What is the date for LENGTH 5?	D	D	M	M	Y	Y	Y	Y
R31	What is the value of LENGTH 5?	☐☐☐ cm							
R32	Is there another weight recorded (LENGTH6)?<br>Do not include todays length	1 = Yes				0 = No<br>➔**SKIP to R35**			
R33	What is the date for LENGTH 6?	D	D	M	M	Y	Y	Y	Y
R34	What is the value of LENGTH 6?	☐☐☐ cm							
R35	What is the length recorded today?	☐☐☐ cm							
R36	CHECK QUESTION: is this baby in the 14 week age group or the 6month age group?	1= 14 week age group<br>➔**SKIP to R39**				2= 6 months age group			
R37	Has the MUAC been recorded on the RTHC?	1 = Yes				0 = No			
R38	What is the child's MUAC today ?<br>Measure the MUAC today using the child MUAC tape	☐☐,☐ cm							
R39	CHECK QUESTION: Is this the mother of the child?	1 = Yes				0 = No<br>➔**SKIP to END**			
R40	What is the weight of the mother today<br>**Isisindo samama namhlanje**	☐☐.☐☐ Kg							
R41	Mothers height today<br>**Ubude bamama namhlanje**	☐☐☐ **cm**							
R42	Mothers MUAC today<br>**I-MAUC kamama namhlanje**	☐☐☐ **cm**							

END - Thank you for taking time to answer these questions.

END- Ngiyabonga ngesikhathi onginike sona sokuphendula lemibuzo
